# Robot-Assisted Thoracic Surgery for Lung Cancer in Juntendo University -Safety and benefit-

**DOI:** 10.14789/ejmj.JMJ25-0040-P

**Published:** 2026-02-13

**Authors:** MARIKO FUKUI, KENJI SUZUKI, YOSHIHIRO ITAGAKI, YUKIO WATANABE, SHUNSUKE UCHIDA, TAKESHI MATSUNAGA, ARITOSHI HATTORI, KOTA IMASHIMIZU, KAZUYA TAKAMOCHI

**Affiliations:** 1 Department of General Thoracic Surgery, Juntendo University, Tokyo, Japan; 1 Department of General Thoracic Surgery, Juntendo University, Tokyo, Japan; 2 Clinical Engineering Unit, Juntendo University, Tokyo, Japan; 2 Clinical Engineering Unit, Juntendo University, Tokyo, Japan

**Keywords:** robotic surgery, lung cancer, safety, advanced surgeries

## Abstract

As of 2017, robotic surgery accounted for less than 1% of lung cancer operations. The adoption of robotic surgery for lung cancer has rapidly increased, now comprising over 15% of such procedures.

Juntendo University initiated robotic thoracic surgery in 2017 and currently performs approximately 250 robotic surgeries annually, including 150 for lung cancer. While robotic surgery offers advantages such as a three-dimensional field of view and enhanced dexterity through multi-jointed instruments, managing intraoperative complications―particularly bleeding―has been considered a limitation. To address these challenges, our hospital has implemented optimized operating room layouts, conducted emergency thoracotomy simulations, and developed safe surgical protocols.

In recent years, the precision of robotic systems has enabled minimally invasive approaches to complex procedures. We have successfully performed advanced surgeries using robotic or robot-assisted techniques, including bronchoplasty, pulmonary artery reconstruction, deep lymph node dissection, and operations involving severe adhesions that are typically difficult with conventional minimally invasive methods.

With continued advancements in robotic surgery, a growing number of patients are expected to benefit from minimally invasive treatment options.

## Introduction

Surgical approaches to lung cancer have diversified significantly with the advancement of minimally invasive techniques. According to the guidelines published by the Japanese Lung Cancer Society^1)^, thoracoscopic lobectomy is strongly recommended and robot-assisted lobectomy is weakly recommended. As of 2017, its use in lung cancer procedures was limited, accounting for less than 1% of cases^2)^. However, following the inclusion of lobectomy under national health insurance in 2018 and segmentectomy in 2020, the adoption of robot surgery has grown substantially. Currently, robotic- assisted procedures are used in over 15% of lung cancer surgeries^3)^.

The efficacy of robot-assisted thoracic surgery (RATS) has been well-documented. Five major randomized controlled trials comparing RATS and video-assisted thoracic surgery (VATS) have been published. Patel YS et al. reported that RATS yielded better health-related quality of life (HRQOL) scores at 12 weeks compared to VATS^4)^. Veronessi G et al.^5)^ and Terra RM et al.^6)^ reported there was no significant difference in hospital stay or complications between RATS and VATS. Jin R et al.^7)^ and Catelli C et al.^8)^ reported RATS offers superior lymph node dissection. Large-scale meta-analyses incorporating these trials have also been conducted^9, 10)^, consistently showing that while RATS is more costly, it is non-inferior in other clinical outcomes.

Nevertheless, RATS has its limitations. Nakamura et al. conducted a questionnaire survey at nine pilot facilities in Japan^11)^. The report states that the advantages of RATS are high image quality, 3D field of view, high mobility with multiple joints, and prevention of tremors. And the disadvantages include difficulty in responding to emergencies, lack of tactile sensation, and the possibility of reduced training opportunities due to solo surgery. Juntendo University adopted RATS to leverage its unique benefits while ensuring procedural safety.

## Clinical practice in robot-assisted thoracic surgery at Juntendo University

**Figure 1:**
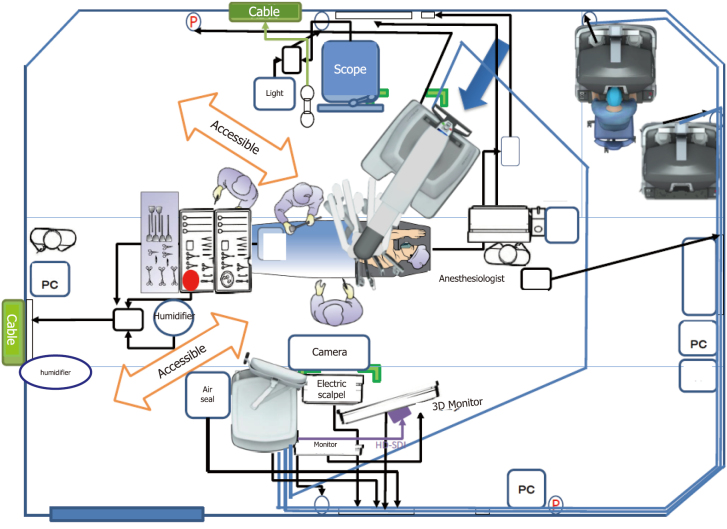
Layout of robotic surgical setup for a left lung tumor. All instrument wiring is routed alongside the anesthesia machine at the head of the patient, ensuring unobstructed access from the caudal side. The Da Vinci patient cart is positioned diagonally toward the patient, creating sufficient space to allow for emergency thoracotomy if needed.

**Figure 2:**
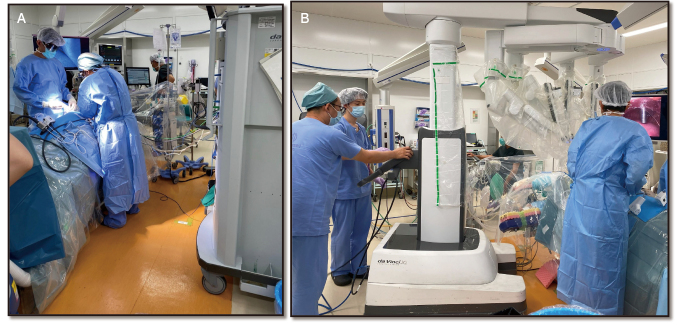
A. View from the caudal side reveals a clear, unobstructed area with no wiring, providing ample space for surgical access. B. To ensure readiness for emergency thoracotomy, a deliberate space is maintained between the patient cart and abdominal area.

At Juntendo University, robot-assisted thoracic surgery (RATS) was introduced in 2017 with careful consideration given to emergency response protocols. The robotic system layout was specifically designed to facilitate rapid access in case of intraoperative emergencies (Figure 1). All surgical instrument wiring is routed through the patient's head, enabling the surgeon to approach from the caudal side with ease (Figure 2A). The robot is positioned diagonally relative to the patient, creating sufficient space for the surgeon to intervene promptly if needed (Figure 2B). One assistant is positioned on each side of the patient to ensure rapid response in the event of bleeding—one is responsible for applying direct pressure to control hemorrhage, while the other is prepared to perform an emergency thoracotomy. Additionally, an emergency incision line is pre-marked in every case to facilitate immediate thoracotomy if required. To further reinforce emergency preparedness, our team conducts scenario-based thoracotomy simulations during key transitions—such as when a new console surgeon is appointed or when the operating room layout is modified. These targeted rehearsals help ensure that all staff members are familiar with emergency protocols and spatial coordination.

**Figure 3:**
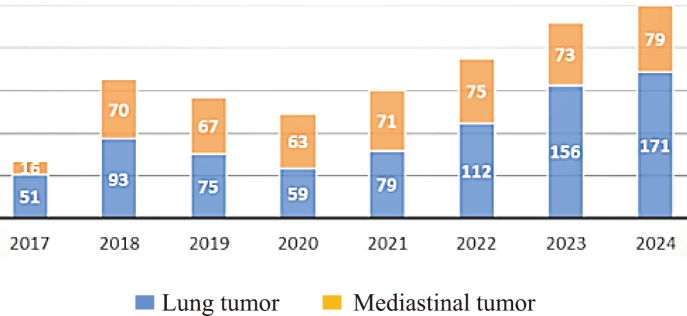
Number of robotic surgeries performed at Department of General Thoracic Surgery in Juntendo University

We also standardize procedures for operations within the thoracic cavity to prevent bleeding and organ damage. During instrument exchanges in robotic surgery, we ensure a wide-angle field of view and operate at angles that minimize the risk of organ injury. Forceps are maneuvered with particular attention to the vertebral bodies, aorta, and heart, avoiding any pressure from the instrument shaft. Care is taken to prevent the forceps from colliding or interfering with one another, thereby avoiding abrupt or uncontrolled movements. Additionally, because the forceps exert a strong gripping force, tissues are handled with gentle pressure when being spread to minimize trauma. By adhering to these safety protocols, the incidence of serious complications has decreased since the introduction of robotic procedures^12)^. To enhance surgical safety, we employ a strategy known as “cool conversion,” which involves transitioning to thoracotomy at an early stage. This proactive approach helps minimize the risk of bleeding caused by adhesions between lymph nodes and blood vessels. With the establishment of robust safety protocols, we were able to increase the number of RATS. In the year RATS was initiated, there were 70 cases annually, but by 2024, the number had increased to 250 cases per year (Figure 3). The indications and contraindications for RATS are not strictly defined and often depend on a combination of patient-specific factors, institutional resources, and surgeon expertise. At our hospital, case selection is based on a comprehensive evaluation of clinical factors, including tumor location, anatomical complexity, comorbidities, and patient preferences. While RATS is generally considered suitable for early-stage lung cancer and mediastinal tumors, we also apply it to selected complex cases when safety can be ensured through preoperative planning and intraoperative adaptability. This flexible approach to case selection has allowed us to explore the full potential of robotic surgery beyond conventional indications.

This approach has made robotic surgery a viable option not only for standard procedures but also for highly advanced and technically demanding operations. The precision and dexterity afforded by robotic systems make them particularly well-suited for complex cases such as sleeve resections^12, 13)^, where meticulous dissection and reconstruction are required. Additionally, robot-assisted techniques enable effective tumor resection in anatomically challenging regions that are difficult to access using conventional approaches^14, 15)^. Furthermore, by integrating robotic surgery with open thoracotomy when necessary, it is now possible to extend the benefits of minimally invasive surgery to cases previously considered unsuitable for such techniques. This includes superior sulcus tumors^16)^, which often involve critical neurovascular structures, and tumors requiring vascular reconstruction^17)^. The hybrid approach allows surgeons to leverage the enhanced visualization and instrument control of robotics while maintaining the flexibility and access of traditional open surgery, thereby expanding the scope of minimally invasive thoracic procedures without compromising safety or oncological outcomes.

Robotic surgery is increasingly being integrated into surgical education, offering unique advantages for training and skill development. One of its key educational benefits is the shared visual field: both trainees and experienced surgeons view the same high-definition, magnified 3D images in real time. This allows trainees to closely observe the expert's perspective, including subtle hand movements, tissue handling techniques, and decision-making processes that are often difficult to convey in traditional open or thoracoscopic procedures. Moreover, the dual-console system enables seamless alternation of control between the instructor and the trainee. This setup allows the supervising surgeon to guide the trainee step-by-step, intervene when necessary, and provide immediate feedback. Instructors can also use on-screen annotations or verbal cues to highlight anatomical landmarks, demonstrate optimal instrument trajectories, and point out areas for improvement in technique. This interactive and immersive learning environment not only enhances technical proficiency but also fosters confidence and precision in surgical maneuvers. By combining advanced visualization, controlled instrument handling, and real-time mentorship, robotic surgery serves as a powerful platform for cultivating the next generation of thoracic surgeons.

## Conclusion

This report summarizes the status of robotic lung cancer surgery in Juntendo University. In lung cancer surgery, "cancer control" and "safety" are important. The widespread use of robotic surgery is expanding the range of minimally invasive surgical procedures available. Robotic surgery also has potential for educational opportunities for young surgeons.

## Author contributions

MF coordinated the institutional implementation of robotic surgery and was responsible for patient data analysis and interpretation. KS supervised all robotic procedures and contributed to manuscript preparation. YI designed the layout of the robotic procedures. All authors read and approved of the final manuscript.

## Conflicts of interest statement

The authors declare that there are no conflicts of interest.
